# Dynamic Viscoelasticity and Surface Properties of Porcine Left Anterior Descending Coronary Arteries

**DOI:** 10.1007/s13239-016-0288-4

**Published:** 2016-12-12

**Authors:** Hanna E. Burton, Jenny M. Freij, Daniel M. Espino

**Affiliations:** Department of Mechanical Engineering, University of Birmingham, Birmingham, B15 2TT UK

**Keywords:** Coronary arteries, Dynamic mechanical analysis (DMA), Loss, Storage, Surface roughness, Viscoelasticity

## Abstract

The aim of this study was, for the first time, to measure and compare quantitatively the viscoelastic properties and surface roughness of coronary arteries. Porcine left anterior descending coronary arteries were dissected *ex vivo*. Viscoelastic properties were measured longitudinally using dynamic mechanical analysis, for a range of frequencies from 0.5 to 10 Hz. Surface roughness was calculated following three-dimensional reconstructed of surface images obtained using an optical microscope. Storage modulus ranged from 14.47 to 25.82 MPa, and was found to be frequency-dependent, decreasing as the frequency increased. Storage was greater than the loss modulus, with the latter found to be frequency-independent with a mean value of 2.10 ± 0.33 MPa. The circumferential surface roughness was significantly greater (*p* < 0.05) than the longitudinal surface roughness, ranging from 0.73 to 2.83 and 0.35 to 0.92 *µ*m, respectively. However, if surface roughness values were corrected for shrinkage during processing, circumferential and longitudinal surface roughness were not significantly different (1.04 ± 0.47, 0.89 ± 0.27 *µ*m, respectively; *p* > 0.05). No correlation was found between the viscoelastic properties and surface roughness. It is feasible to quantitatively measure the viscoelastic properties of coronary arteries and the roughness of their endothelial surface.

## Background

Coronary heart disease is the leading cause of mortality worldwide.^99^ In the USA, for example, coronary heart disease was the underlying cause of death in 1 out of every 7 deaths in 2011 and direct and indirect costs associated with heart disease were estimated to be $204.4 billion in 2010.^80^ This study has determined, and compares, the viscoelastic and surface roughness properties of coronary arteries, specifically along the left anterior descending (LAD) artery. Characterisation and quantification of these properties of coronary arteries is important for the development of clinical treatments through novel designs of vascular implants (e.g. stents and grafts) and tissue engineered replacements.^51^

Coronary artery disease can lead to chronic narrowing of the vessels or impaired vascular function, which can increase the risk of a myocardial infarction.^63^ The LAD artery is part of one of the two major branches of the coronary circulation,^51^,^63^ supplying oxygenated blood to the ventricular myocardium. It also supplies the left atrium, left atrial appendage, pulmonary arteries and aortic root.^61^

Material properties of coronary arteries have been characterised for both human^58^,^61^,^86^,^102^ and porcine^68^,^102^,^104^ arteries. Uniaxial tests have been performed on coronary arteries to calculate tensile strength^17^ and Young’s modulus^42^,^58^ of the material. Tensile tests have been performed on separate layers of the coronary artery (intima, media and adventitia).^51^ Material properties, though, are dependent on direction,^108^ with stress in the circumferential ‘direction’ (i.e. aligned with the circumference of the artery) being greater than that longitudinally (i.e. along the length of the artery). Thus, biaxial testing has been of interest with stress–strain characterisation of, for example, diseased coronary arteries.^65^ To further maintain the physiological state, pressurised tests have been used to measure deformation of vessels that allow characterisation of the stress–strain relationship. Such tests have been used to calculate the elasticity of coronary arteries.^103^ A typical assumption for most tests is that coronary arteries are incompressible, with Karimi *et al.* recently measuring the Poisson’s ratio of both healthy and atherosclerotic human coronary arteries to justify this assumption.^59^ These studies have quantitatively measured mechanical properties but they ignore the intrinsic viscoelasticity of the coronary artery.

Most soft connective tissues are viscoelastic, including the coronary arterial walls.^19^,^41^,^49^ Hence, the stress–strain relationship is a function of the loading rate.^110^ Changes in the viscoelastic properties of arteries are apparent in patients with vascular disease.^98^ The viscoelastic properties of arteries which have been studied, include hysteresis loops of a cross-sectional area as a function of pressure for large ovine arteries,^101^ creep and stress relaxation of porcine carotid arteries,^10^ creep of human coronary arteries,^86^ and stress relaxation in the longitudinal and circumferential direction of porcine carotid arteries.^34^ However, the effect of dynamic viscoelastic properties of the coronary arteries, using dynamic mechanical analysis (DMA), has not been quantified.

DMA enables a material’s viscoelastic properties to be determined at physiologically relevant frequencies. The viscoelastic properties are characterised by storage and loss moduli^53^ which describe the material’s ability to store and dissipate energy, respectively. DMA has been applied to soft connective tissues, including articular cartilage,^32^,^92^ intervertebral discs,^33^ chordae tendineae^107^ and the bladder tumours.^6^ An advantage of quantifying viscoelastic properties by a dynamic method, over conventional stress relaxation and creep methods, is that the physiological loading conditions can be more closely replicated.^69^ For example, it enables frequency-dependent viscoelastic properties to be characterised. The frequency-dependent relationship of dynamic elastic modulus has been investigated through a pressurised model of mice pulmonary arteries.^105^ However, characterisation of frequency-dependency has not been performed for coronary arteries, let alone for their dynamic viscoelasticity.

Changes to blood vessels such as stenosis (narrowing of arteries), calcification or damage of the endothelial surface can be disruptive to the blood flow and lead to further clinical complications.^11^,^82^ The changes seen can indicate signs of disease of the arteries, such as atherosclerosis.^44^ However, assessment of changes to the surfaces of vessels have been qualitative.^11^ If stents or biomaterials are to be designed to mimic natural surfaces, for example by nano-texturing,^83^ or grafts designed to encourage endothelialisation,^56^,^73^,^93^,^109^ then it is necessary to quantify the properties of the surface of healthy coronary arteries. Surface roughness can be quantified using *Ra* (the arithmetic average of absolute values of sampling length). Although its applications are mainly in tribology and wear,^21^,^60^ it has recently been used to study biological tissues such as articular cartilage in order to assess its surface roughness.^35^,^88^ It has also been trialled for cardiovascular applications, not involving tribology. For example, red blood cells have been studied at a nano-scale through the use of atomic force microscopy,^4^ and average roughness values compared after treatment of cells through fixation and staining.^36^ Further, the roughness of blood cells can be used as an indication of the health of cells, where red blood cells of diabetics appear smoother than cells from non-diabetics.^14^

The aims of this study were to characterise the frequency-dependent longitudinal viscoelastic properties and surface roughness of the LAD coronary artery in porcine hearts. The variation of viscoelastic properties and surface roughness were assessed along the length of the LAD artery. The relationship between viscoelastic and surface roughness properties was also analysed. Porcine hearts were used for this study as they are an established model of the human heart based on their anatomical similarity.^86^,^102^

## Methods

### Specimens

Eight porcine hearts were supplied by Fresh Tissue Supplies (Horsham, UK). Hearts were frozen on excision. After delivery to the laboratory, the hearts were wrapped individually in tissue paper soaked in Ringer’s solution. They were then stored in heat sealed bags at −40 °C, following protocols from previous studies of porcine heart tissue.^22^,^23^,^25^,^77^

**Figure 1:**
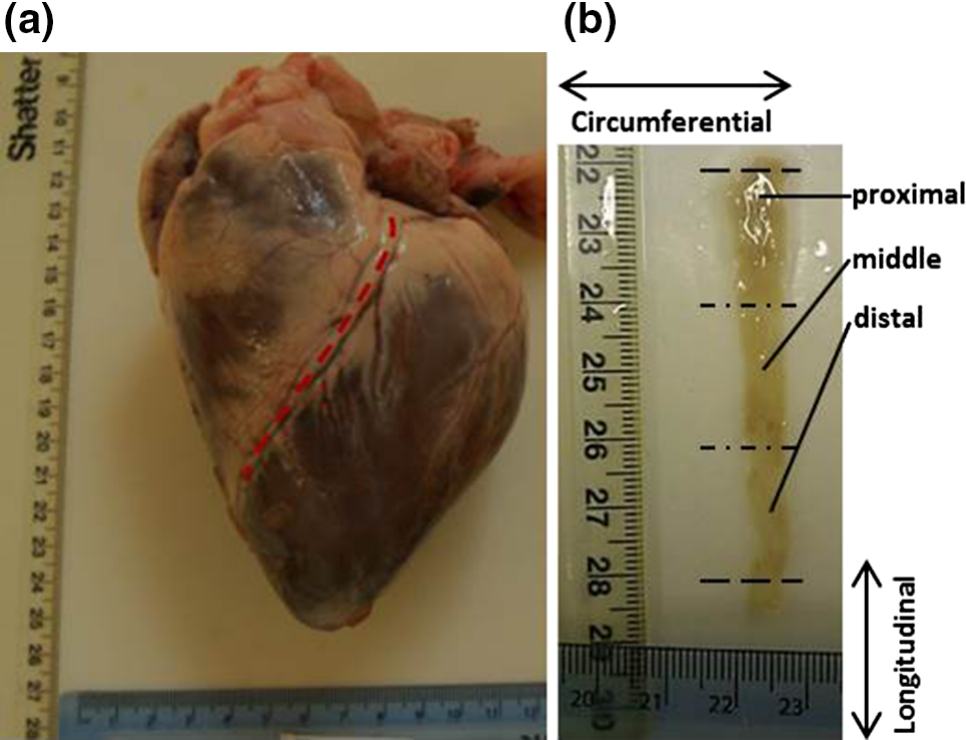
Porcine heart and LAD artery. (a) LAD coronary artery highlighted by a dotted line on the porcine heart. (b) LAD artery sample dissected and cut open to reveal the endothelial layer; longitudinal and circumferential orientations are highlighted.

Hearts were defrosted at approximately 4 °C overnight before dissection. The LAD artery was identified on the heart (Fig. 1a), and dissection of the LAD artery was performed starting from the most distal point visible to the bifurcation of the LAD and left circumflex artery. Eight LAD artery samples were obtained in total, one from each heart. These were cut open longitudinally (i.e. along the long-axis of the artery). Care was taken not to damage the endothelial surface away from the incision. Excess cardiac muscle tissue was removed from samples leaving coronary artery tissue only (Fig. 1b).

Starting at the most proximal position of each LAD artery, the samples were sectioned into three pieces of approximately 20 mm in length: the proximal, middle and distal pieces (Fig. 1b). The remainder of the LAD artery was discarded. Specimens were wrapped in tissue paper soaked in Ringer’s solution (Oxoid Ltd, Bassingstoke, UK) and stored in heat sealed bags at −40 °C ready for subsequent testing. Before testing, samples were defrosted overnight at approximately 4 °C.

### Mechanical Testing

#### Dynamic Mechanical Analysis

A Bose ElectroForce 3200 (Bose Corporation, ElectroForce Systems Group, Minnesota, USA) testing machine operated with WinTest DMA software (Bose Corporation, ElectroForce Systems Group, Minnesota, USA) was used to perform DMA on the tissue specimens. The use of this method to determine viscoelastic properties of natural tissues is explained further elsewhere.^7^,^32^ Briefly, a Fourier analysis of the force and out-of-phase displacement waves were performed and the magnitude of the force, the magnitude of the displacement, phase lag, *δ* and frequency were determined.^69^ From this, the complex stiffness, k* (ratio of magnitude of the force to magnitude of the displacement) was calculated. Using the shape factor for a rectangular test specimen, *S* (Eq. 1),^76^ the storage (*E*′) and loss (*E*″) moduli were calculated using Eqs. (2) and (3), respectively.

1 $$S = \frac{wt}{l}$$

2 $$E^{\prime} = \frac{{k^{*} \cos (\delta )}}{S}$$

3 $$E^{\prime\prime} = \frac{{k^{*} \sin (\delta )}}{S}$$

Here, *w* is width, *t* is thickness and *l* is length of the specimen sample.

#### Experimental DMA Protocol

**Figure 2:**
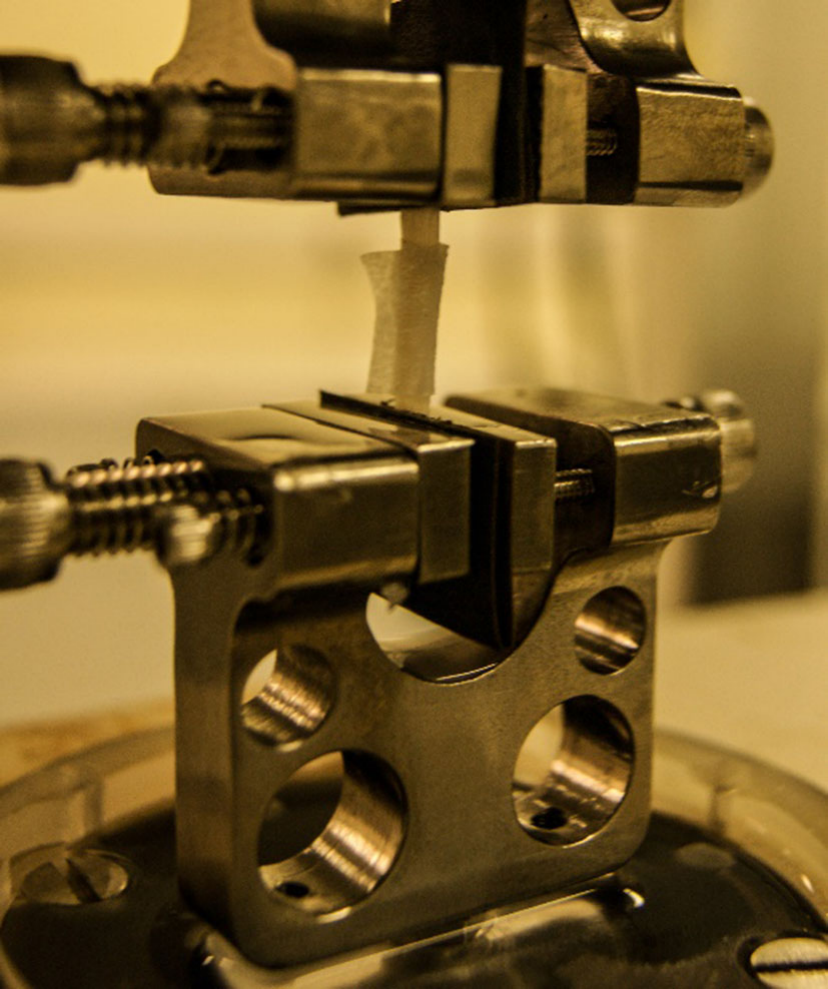
The specimen setup between the gripers, wrapped in tissue paper soaked in Ringer’s solution and pre-loaded.

Specimens were held in place for testing using grips lined with emery paper leaving an un-stretched gauge length of 4.57 ± 0.75 mm. The gripping method, shown in Fig. 2, is similar to that used by other studies.^77^,^107^ Samples were preloaded to a stretched gauge length of 16.63 ± 2.24 mm to remove the slack seen by the tissue due to dissection from surrounding tissue, as the full length of samples before dissection (gauge plus gripping length) was 20 mm. Coronary arteries require preconditioning;^5^,^64^ therefore, samples were loaded at a rate of 0.5 mm/s and stretched by 3 mm for ten cycles.

For testing, two sinusoidal displacement loading protocols were used. One between 1 and 1.5 mm and the other between 1.5 and 2 mm, i.e. mean displacement of 1.25 and 1.75 mm, respectively. Both loading protocols used a dynamic amplitude of 0.50 mm (i.e. peak to trough). Previous studies measured the median longitudinal displacement of the LAD artery to be 1.36 mm.^5^ Therefore, tests between 1 and 1.5 mm correspond to the median longitudinal displacement. The 1.5 and 2 mm range, instead, corresponded to coronary arteries undergoing deformation above median values, also reported in literature.^5^

Samples were loaded over frequencies ranging from 0.5 Hz up to 10 Hz. Between 0.5 and 5 Hz, testing frequencies increased at 0.5 Hz intervals; whereas, from 6 to 10 Hz testing frequencies increased in 1 Hz increments. The frequency range covered bradycardia (<60 beats per minute, bpm; i.e. less than 1 Hz), physiological heart rates (from 1 up to 3 Hz for exercise), and tachycardia (>250 bpm, i.e. 4 Hz).^43^ The frequencies up to 10 Hz were estimated to represent the maximum strain rates for coronary arteries of the left ventricle.^67^ All mechanical testing was performed at room temperature.

### Tissue Preparation

Following mechanical testing, tissue specimens were stored at −40 °C, and before processing they were defrosted (as described in the "Specimens" section, above). Specimens underwent fixation to enable surface imaging. A standard protocol for fixation of soft mammalian tissues was followed.^8^ Briefly, tissue specimens (Fig. 1b) were fixed in 3% glutaraldehyde solution (Fluka Analytical, Sigma Aldrich, St Louis, MO, USA) in 0.2 M sodium phosphate buffer for 1 h.

The samples underwent dehydration using a series of washes for 10 min each, with ethanol (Fisher Chemical, Fisher Scientific UK Ltd, Loughborough, UK) concentrations at 30, 50, 70, 95 and 2 × 100%. Hexamethyldisilazane (HMDS) (Aldrich Chemistry, St Louis, MO, USA) was then used to complete dehydration.^78^ The samples were placed in an HMDS wash for 15 min, before replenishing with fresh HMDS and being left to evaporate overnight.

### Surface Imaging

Optical imaging was performed using an Alicona microscope (G5 Infinite Focus, Alicona UK, Kent, UK). Processed tissue specimens were scanned at ×10 magnification (10× Nikon CFI 60 TU Plan Epi Infinity Corrected Obj lens, Alicona UK, Kent, UK). This was chosen as an appropriate magnification as the minimum measurable *Ra* (0.3 *µ*m) of the ×10 magnification for the microscope was optimum for the range of measured *Ra* data during preliminary testing.^3^ Scanning was performed between the maximum and minimum focussing positions of the *z* height of each sample surface through focussing of the lens. Similarly, the area of the scan was controlled by selecting the maximum and minimum *x* and *y* positions of the sample. Note, the *x* and *y* axes are parallel to the circumferential and longitudinal directions, respectively (Fig. 1b), and the *z* axis is perpendicular to the *x*–*y* plane (i.e. aligned parallel to the direction of the thickness of the sample).

**Figure 3:**
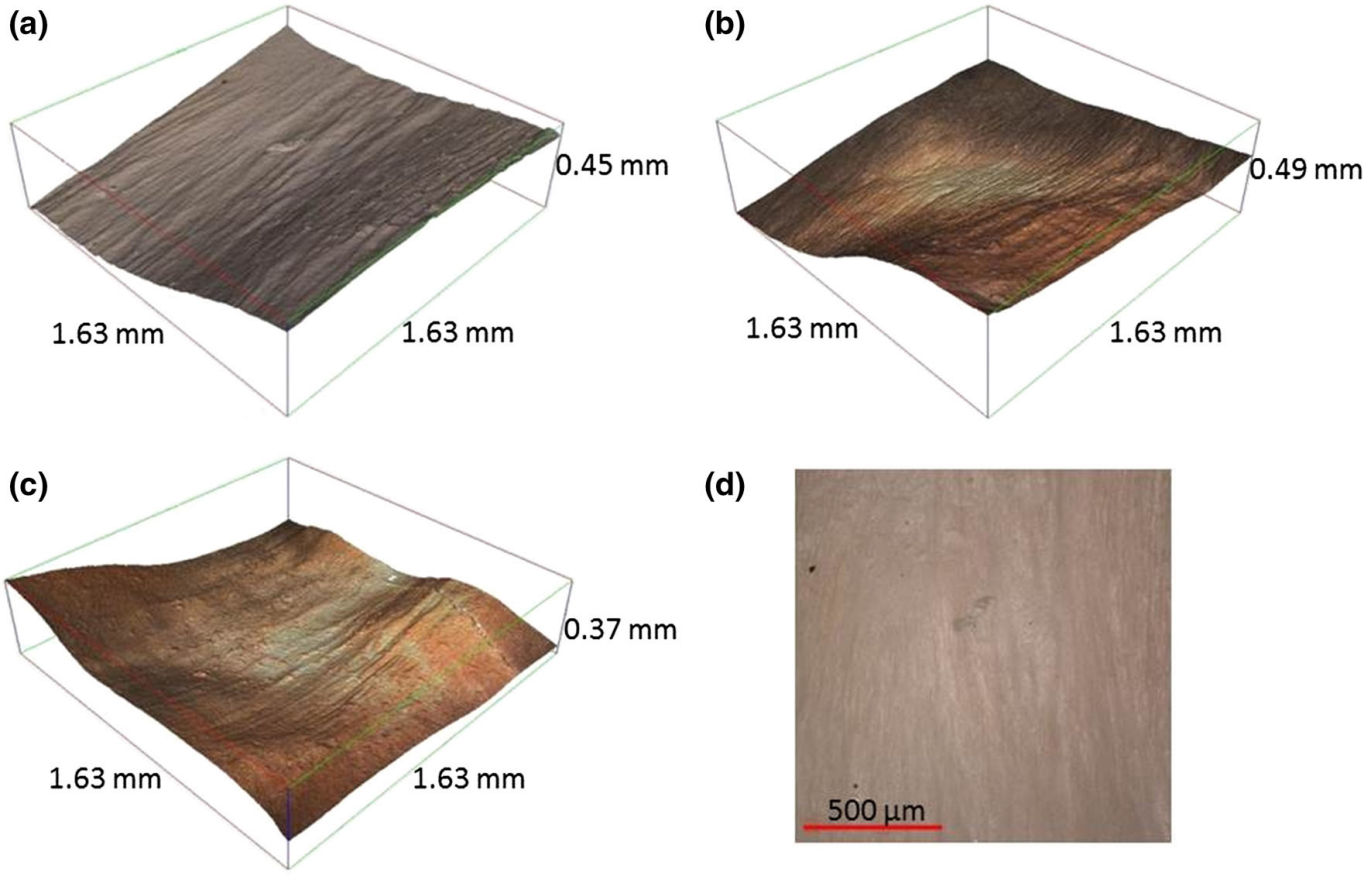
Three dimensional reconstruction of the endothelial surface of the LAD (×10). Ridges are observable across the circumferential direction (grooves appearing in longitudinal direction). Reconstructed surfaces at (a) proximal, (b) middle and (c) distal positions, and (d) optical 2D image of a proximal specimen.

The Alicona IF-Laboratory Measurement Module (version 6.1, Alicona UK, Kent, UK) generates a three-dimensional (3D) point cloud by using contrast based focus detection and focus stacking to calculate the depth of microscopy images. This method has been shown to be comparable to traditional methods, such as scanning electron microscopy, for measuring surface roughness.^95^ The 3D point cloud represented as a reconstructed surface is shown in Fig. 3. The 3D models were used to measure *Ra*, consistent with other studies.^1^,^100^ *Ra* was measured along the lengths of the reconstructed images in both the longitudinal direction of the artery, *Ra*_L_, and across the circumference of the artery, *Ra*_CU_, (Fig. 1b). *Ra*_CU_ was calculated using Eq. (4); *Ra*_L_ was calculated using Eq. (5).^54^ For each specimen, five repeat measurements were taken for both *Ra*_L_ and *Ra*_CU_. When measuring *Ra*, care was taken to avoid areas that had been damaged by clamping when specimens were gripped during the mechanical testing. The edges of the sample where distortion may have been caused due to dissection or processing were also avoided. Finally, bifurcation ‘holes’ where smaller vessels connected to the LAD artery were not imaged as it forms part of the blood vessel structure rather than being an intrinsic property of the surface.

4 $$Ra_{\text{CU}} = \frac{1}{l}\mathop \int \limits_{0}^{l} |Z\left( x \right)|dx$$

5 $$Ra_{\text{L}} = \frac{1}{l}\mathop \int \limits_{0}^{l} |Z\left( y \right)|dy$$

Here, *l* is measured sample length, *Z*(*x*) is height of profile at position *x*, and *Z*(*y*) is height of profile at position *y*. Following preliminary analysis, Eq. (6), quantifying the average shrinkage value of tissue in the circumferential direction, was used to correct *Ra*_CU_ for tissue shrinkage during tissue processing; whereas, *Ra*_L_ was not corrected as it was not altered by the tissue processing.

6 $$Ra_{\text{C}} = \frac{{Ra_{\text{CU}} }}{(1 - 0.63)}$$

Here, *Ra*_C_ is the corrected value, and *Ra*_CU_ is the uncorrected raw value measured initially.

### Data Analysis and Statistics

In total, *n* = 720 frequency data points were analysed for DMA (i.e. 8 LAD arteries × 3 specimens per artery × 2 displacements × 15 frequencies). Of these data points, *n* = 35 points were not recorded by the WinTest software due to machine error during testing.

Pierce’s criterion was applied across the frequencies to highlight possible outlier data. For the *n* = 84 points highlighted, data was re-analysed manually. Of these points, *n* = 15 data points saw irregular noise across the sample wave. Where possible the wave was ‘smoothed’ to remove noise, but for *n* = 4 data points the noise to signal ratio remained large and prevented analysis, i.e. these four data points were deleted. Although the remaining *n* = 69 data points had high levels of background noise, manual analysis demonstrated that a sample wave suitable for analysis was available. The larger than normal disparity was treated as being due to natural variation seen in biological tissues. Therefore, a total of *n* = 681 (out of 720) data points were used for data analysis.

Data analysis was performed using SigmaPlot 12.0 (Systat Software Inc., London, UK). All data sets were assessed for normal distribution using a Shapiro–Wilk normality test. If data sets were normally distributed (i.e. *p* > 0.05), significance was assessed (*p* ≤ 0.05) using a paired *t* test. If data was not normally distributed, a Wilcoxon Signed Rank test was used (i.e. a paired non-parametric test; *p* ≤ 0.05 for significance).^12^,^90^

Unless otherwise stated, the paired comparisons used moduli results at 1 Hz, with an extension range of 1–1.5 mm. Paired comparisons included the following:

- moduli frequency-dependency, i.e. 1 vs. 10 Hz;
- variation of moduli between proximal and distal sections;
- extension range influence on moduli, i.e. 1–1.5 vs. 1.5–2 mm;
- *Ra*_C_ against *Ra*_L_ at proximal, middle and distal positions along the LAD artery (and *Ra*_CU_ against *Ra*_L_ for the same positions);
- *Ra*_C_, *Ra*_CU_ and *Ra*_L_ between the proximal and distal sections.

Regression analysis was performed for moduli against frequency, as well as for both the *Ra*_C_ and *Ra*_L_ against storage and loss moduli. Circumferential surface roughness values were evaluated for both *Ra*_CU_ and *Ra*_C_.

## Results

### Viscoelastic Properties

#### Frequency Dependency

The range of storage moduli varied from 14.47 to 25.82 MPa, whereas loss varied from 1.57 to 2.71 MPa (Table 1). The storage modulus was around an order of magnitude greater than the loss modulus.

**Table 1:** Storage and loss moduli of LAD coronary arteries at 1–1.5 mm extension.

Frequency (Hz)	Proximal				Middle				Distal			
	Storage modulus (MPa)		Loss modulus (MPa)		Storage modulus (MPa)		Loss modulus (MPa)		Storage modulus (MPa)		Loss modulus (MPa)	
	Mean	SD	Mean	SD	Mean	SD	Mean	SD	Mean	SD	Mean	SD
0.5	17.27	11.00	1.80	0.99	22.83	6.13	2.23	0.49	25.67	8.85	2.28	0.82
1	16.99	9.74	1.76	0.83	22.78	5.74	2.16	0.51	25.25	8.55	2.26	0.78
1.5	16.78	9.66	1.69	0.80	22.64	5.74	2.14	0.52	25.03	8.37	2.27	0.80
2	16.64	9.66	1.68	0.72	22.52	5.67	2.16	0.52	24.81	8.29	2.26	0.86
2.5	16.49	9.55	1.62	0.79	22.53	5.74	2.14	0.48	24.70	8.46	2.40	0.91
3	16.35	9.48	1.64	0.76	22.13	5.88	2.11	0.46	25.82	8.51	2.38	0.87
3.5	16.25	9.46	1.62	0.76	22.01	5.71	2.15	0.50	25.56	8.55	2.33	0.82
4	16.14	9.41	1.59	0.65	21.79	5.84	2.15	0.57	25.26	8.35	2.41	0.93
4.5	16.03	9.37	1.66	0.74	21.69	5.67	2.12	0.50	25.01	8.36	2.34	0.84
5	15.89	9.37	1.57	0.71	21.43	5.69	2.22	0.58	24.58	8.30	2.41	0.93
6	15.65	9.40	1.68	0.77	20.96	5.66	2.31	0.52	22.76	7.85	2.56	0.93
7	15.43	9.38	1.70	0.76	20.59	5.64	2.32	0.59	23.23	7.98	2.71	1.07
8	15.09	9.43	1.76	0.85	19.74	5.77	2.34	0.57	22.19	7.77	2.60	0.93
9	14.84	9.36	1.78	0.80	19.35	5.50	2.34	0.54	21.29	7.67	2.61	0.90
10	14.47	9.35	1.73	0.76	18.47	5.57	2.23	0.49	19.99	7.31	2.28	0.82

SD, standard deviation

**Figure 4:**
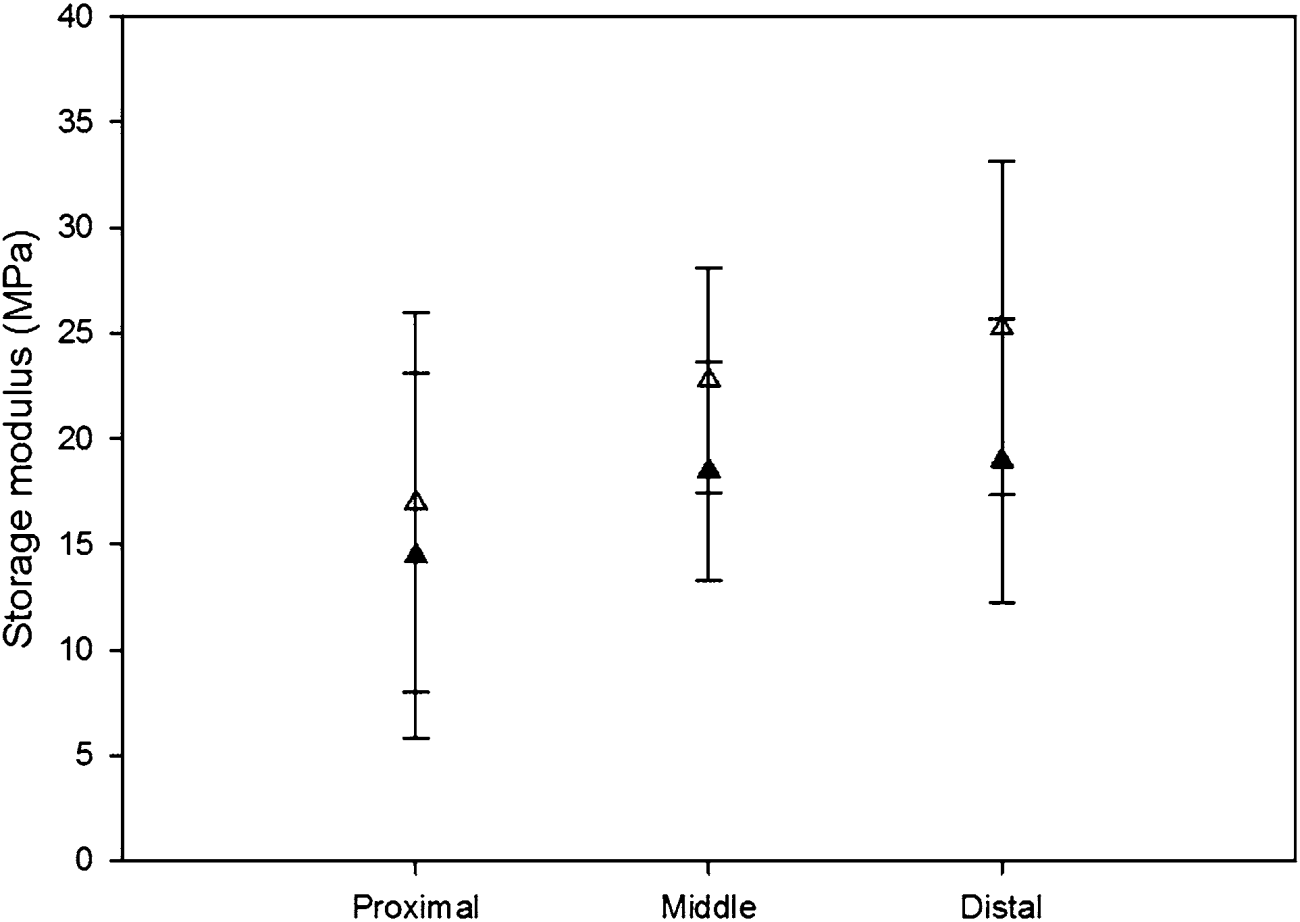
Storage modulus comparison at 1 (white triangles) and 10 Hz (black triangles) for averaged values of proximal, middle and distal specimens. Error bars represent 95% confidence intervals where *n* = 7.

**Figure 5:**
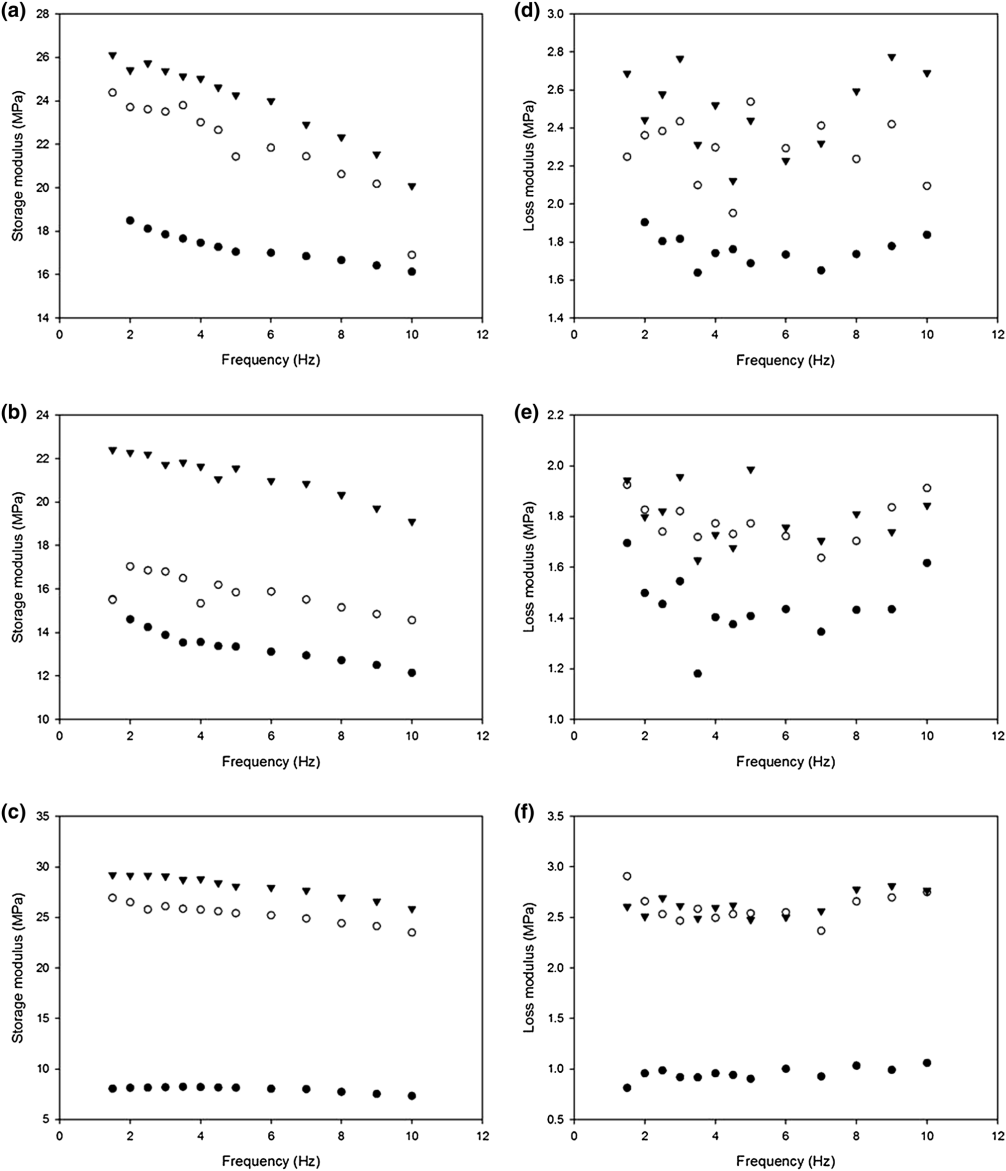
Frequency dependency of storage, (a)–(c), and loss, (d)–(f), moduli for three individual samples (nine specimens): (from top to bottom) Sample 2, 3 and 5. (Black dots for proximal, white dots for middle, black triangles for distal samples).

**Figure 6:**
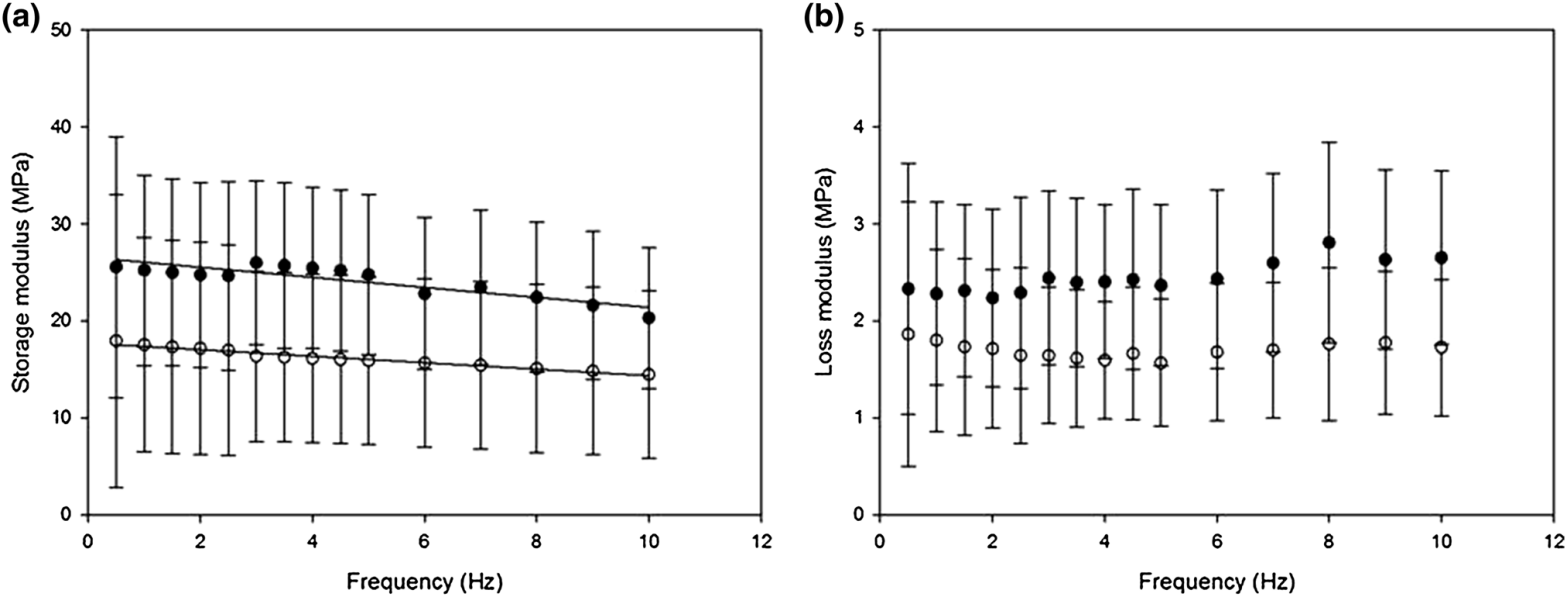
Frequency-dependency of proximal (white dots) and distal (black dots) positions along LAD artery—(a) mean storage and (b) loss moduli. Linear regression lines showing frequency dependent relationship. Error bars represent 95% confidence intervals, where at: 1 Hz, *n* = 5; 1–2.5 Hz, *n* = 6; 3–10 Hz, *n* = 7.

Between 1 and 10 Hz, there was a statistically significant difference in the storage modulus (*p* < 0.05). The modulus at 1 Hz was consistently higher than at 10 Hz, on average by 4.37 MPa (Fig. 4). This was the case at all positions along the LAD artery (i.e. proximal, middle and distal). Figure 5 shows results for individual specimens, demonstrating that the trend was consistent across samples and that it was not skewed by any given individual outlier sample. A linear relationship was found (Fig. 6a), defined by Eq. (7).

7 $$E^{\prime} = mf + c$$

Here, *E*′ is the storage modulus, *f* is frequency, and *m* and *c* are constants (Table 2).

**Table 2:** Regression constants, *m* and *c*, for the dependency of the storage modulus with frequency for average mean of all samples at different positions along LAD artery (*p* < 0.05 for all trends).

Position	*M* (MPa s)	*C* (MPa)	*R* ^2^
Proximal	−0.27	17.24	0.99
Middle	−0.45	23.45	0.97
Distal	−0.54	26.52	0.82

The loss modulus was found to be frequency-independent. No significant differences were found for the loss modulus at 1 and 10 Hz (*p* > 0.05; Table 1). The loss modulus was found to have a mean (±standard deviation) of 1.68 ± 0.07, 2.21 ± 0.09 and 2.41 ± 0.14 MPa for the proximal, middle and distal samples respectively. Individual specimen results are provided in Fig. 5; average proximal and distal values shown in Fig. 6b.

#### Proximal vs. Distal

Storage modulus did not vary along the LAD artery. Although the proximal mean storage modulus was lower than the distal modulus on average by 8 MPa, no significant difference was found between the storage modulus of proximal and distal samples; mean ± SD: 16.99 ± 9.74 MPa (proximal), 25.25 ± 8.55 MPa (distal) (*p* > 0.05). This can be interpreted in Fig. 6a as a difference in the means but with overlap of confidence intervals (due to natural variability).

The loss modulus did not vary along the LAD artery. No significant difference was found between the loss modulus of proximal and distal samples (*p* > 0.05). However, the proximal mean loss modulus was lower than the distal modulus on average by 0.74 MPa, with extensive overlap of 95% confidence intervals (Fig. 6b).

#### Extension Ranges

**Figure 7:**
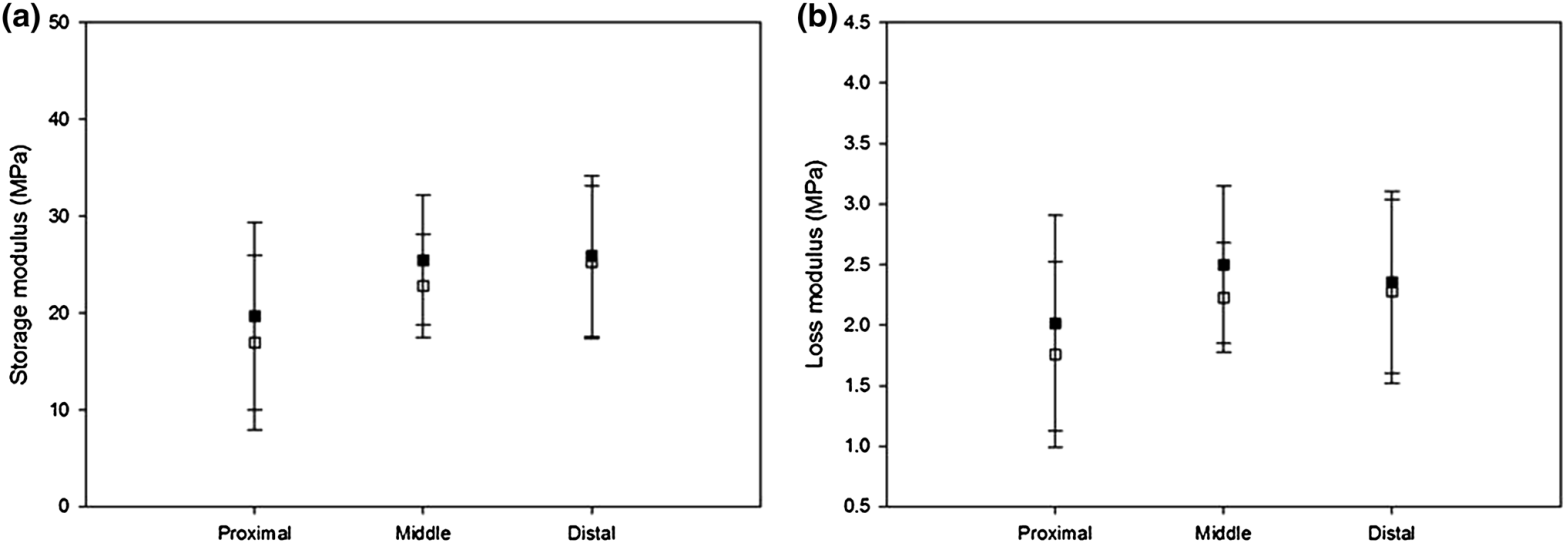
Comparison of viscoelastic properties at 1 Hz for different extension ranges for averaged values of proximal, middle and distal samples—(a) Storage modulus and (b) loss modulus. (White squares for 1–1.5 mm, black squares for 1.5–2 mm). Error bars represent 95% confidence intervals where *n* = 7.

No statistical significance was seen for the storage and loss moduli between extensions of 1–1.5 and 1.5–2 mm. At 1 Hz, testing samples between 1.5 and 2 mm led to an average storage modulus of 19.71, 25.46 and 25.87 MPa for proximal, middle and distal samples, respectively (Fig. 7a). These storage moduli were not significantly different to the storage moduli when measured at 1–1.5 mm of extension (16.99, 22.78 and 25.25 MPa, respectively; *p* > 0.05, Fig. 7).

At 1 Hz, testing samples between 1.5 and 2 mm led to an average loss modulus of 2.01, 2.50 and 2.35 MPa for proximal, middle and distal samples, respectively (Fig. 7b). These loss moduli were not significantly different to the loss moduli when measured at 1–1.5 mm of extension (1.76, 2.23 and 2.28 MPa, respectively; *p* > 0.05, Fig. 7).

### Surface Roughness

#### Circumferential vs. Longitudinal Direction

**Figure 8:**
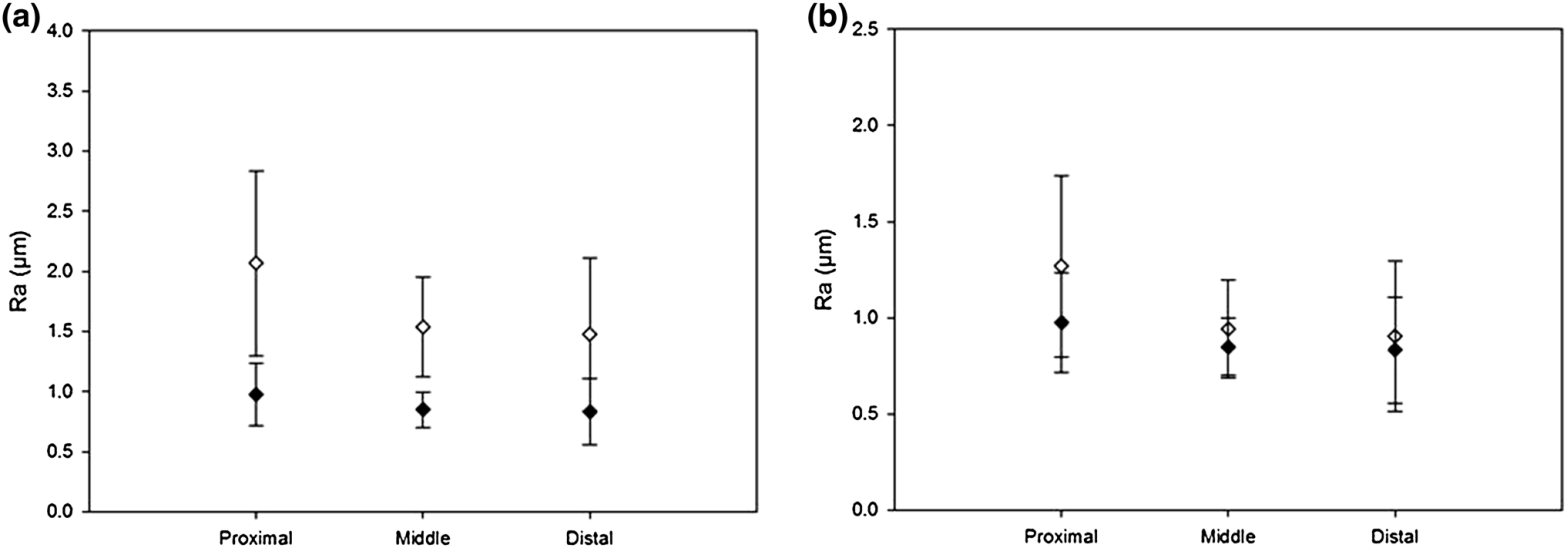
Mean surface roughness (*Ra*) of the proximal, middle and distal LAD coronary artery. Black diamonds for *Ra*_L_ and white diamonds for (a) *Ra*_CU_ and (b) *Ra*_C_. Error bars represent 95% confidence intervals where *n* = 8.

For uncorrected data, LAD arteries were significantly rougher along their circumference than along their longitudinal alignment (*Ra*_CU_ > *Ra*_L_; *p* < 0.05, Table 3). This was the case for proximal, medial and distal samples along the LAD artery (Fig. 8). *Ra*_CU_ ranged from 0.73 to 2.83 *µ*m, and *Ra*_L_ ranged from 0.35 to 0.92 *µ*m. Ridges were observed along the circumferential direction of the LAD artery, but not longitudinally (Fig. 3), consistent with the higher *Ra*_CU_ values compared to *Ra*_L_. However, there was no statistically significant difference between *Ra*_C_ and *Ra*_L_ (*p* > 0.05, Table 3). The corrected *Ra*_C_ ranged from 0.51 to 2.24 *µ*m (Fig. 8), a larger range compared to *Ra*_L_, due to the variation in measurements taken along the circumferential direction.

**Table 3:** Descriptive statistics of surface roughness for *Ra*_CU_ and *Ra*_C_.

Position	Normally distributed?	—*Uncorrected*—						—*Corrected*—		
		Mean (*µ*m)	Median (*µ*m)	SD (*µ*m)	Mean (*µ*m)	Median (*µ*m)	SD (*µ*m)	Mean (*µ*m)	Median (*µ*m)	SD (*µ*m)
		*Ra*_CU_	*Ra*_CU_	*Ra*_CU_	*Ra*_L_	*Ra*_L_	*Ra*_L_	*Ra*_C_	*Ra*_C_	*Ra*_C_
Proximal	Yes	2.07	1.74	0.92	0.97	0.89	0.31	1.27	1.07	0.56
Middle	Yes	1.53	1.44	0.50	0.85	0.83	0.18	0.94	0.88	0.30
Distal	No	1.48	1.24	0.77	0.83	0.73	0.33	0.90	0.76	0.47

#### Variation Along LAD Artery

No statistical difference was found in the variation of circumferential roughness of *Ra*_CU_ or *Ra*_C_, along the length of the artery (proximal vs. distal, *p* > 0.05; Fig. 8). This was also the case for *Ra*_L_ (Table 3). Although both *Ra*_CU_ and *Ra*_C_ of the proximal samples had a higher mean value compared to that of the middle and distal samples (Table 3), the medians were similar to each other (*Ra*_CU_ median ± SD: proximal = 1.74 ± 0.92 *µ*m; middle = 1.44 ± 0.50 *µ*m, distal = 1.24 ± 0.77 *µ*m) (*Ra*_C_ median ± SD; Table 3). This supports the finding of no statistical difference between the results.

### Regression Analysis Between Viscoelasticity and Surface Roughness

**Figure 9:**
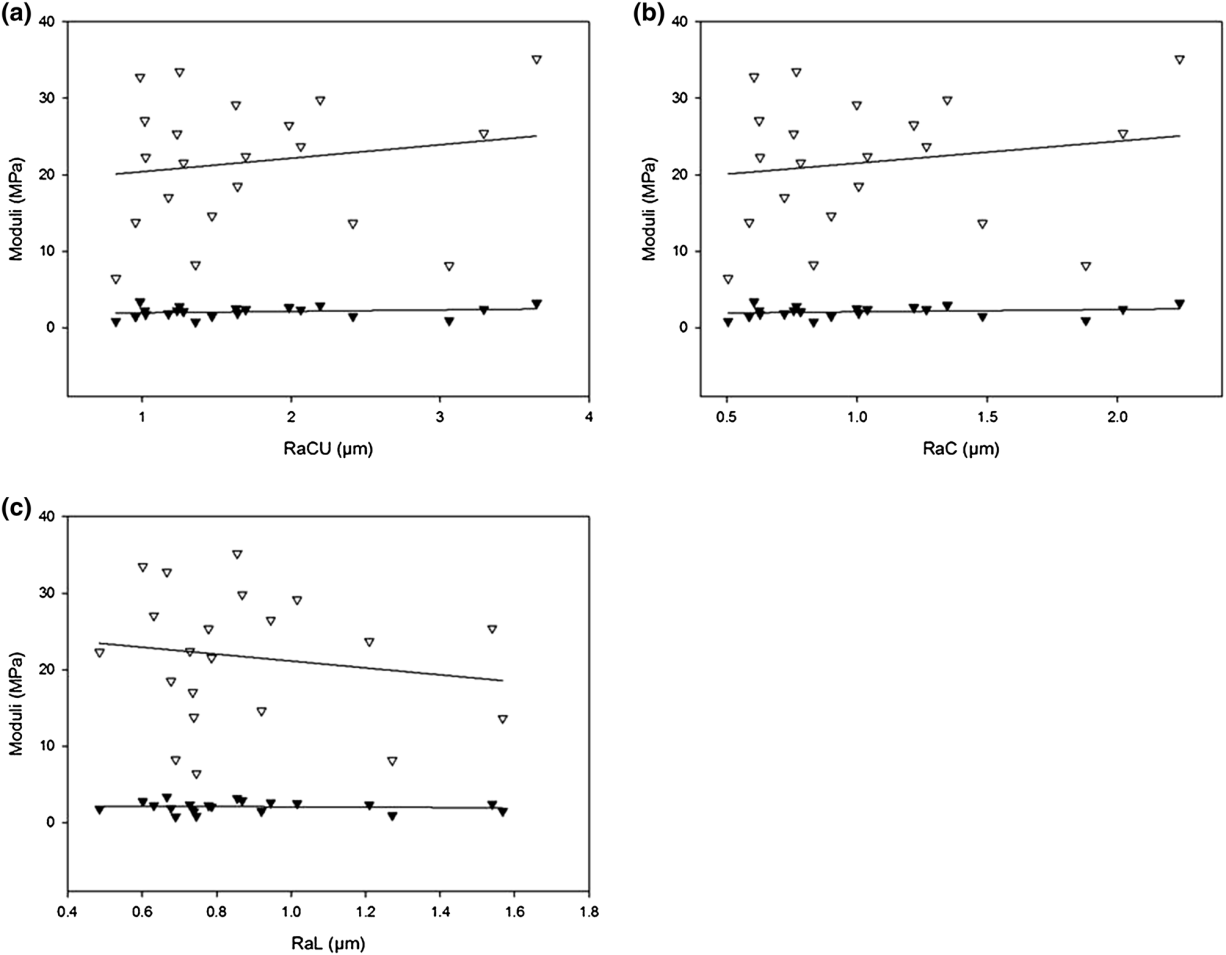
Regression analysis between *Ra*_C_ and viscoelastic properties for (a) *Ra*_CU_, (b) *Ra*_C_ and (c) *Ra*_L_ data against moduli for 1 Hz and 1–1.5 mm extension range (Black triangles for storage, white triangles for loss moduli; *R* ^2^ < 0.05, *p* > 0.05)

Viscoelastic properties and surface roughness were not correlated to each other. For example, at 1 Hz, no correlation was found between *Ra*_C_ and either the storage or the loss moduli. This was the case for both *Ra*_CU_ and *Ra*_C_ (Figs. 9a and 9b). Likewise, *Ra*_L_ was not correlated to either storage or loss moduli (Fig. 9c).

## Discussion

### Findings

To the authors’ knowledge, the frequency-dependent viscoelastic properties and surface roughness of porcine LAD artery have been quantitatively characterised for the first time. The storage modulus was frequency-dependent, whereas the loss modulus was frequency-independent. Storage modulus was found to be much greater than loss modulus. Viscoelastic properties did not vary along the length of the LAD artery. Surface roughness was measured circumferentially and longitudinally. Surface roughness did not vary along the length of the sample. For uncorrected surface roughness data, circumferential roughness was significantly greater than that longitudinally; however, for corrected data there was no significant difference between the two. No correlation was found between the surface roughness and viscoelasticity; given the absence of disease, this was not unexpected. However, there may be benefits in assessing a correlation between the two during disease and this study demonstrates that both can be quantitatively measured.

Frequency-dependent viscoelastic properties of porcine coronary arteries have not previously been quantified. However, viscoelastic properties have been measured in studies of other arteries. The results from this study show that the storage modulus is significantly greater than the loss modulus (approximately ×10) which is consistent with previous results on human arteries, where storage moduli were approximately ×5 larger than loss moduli. The former was approximately 1 MPa as compared to 0.2 MPa for the loss modulus of carotid arteries. Further, the storage modulus varied from 3 to 7 MPa as compared to 0.4–1.6 MPa for femoral arteries.^71^ Similarly, for canine femoral artery the storage was greater than loss modulus (1.20 MPa compared to 0.14 MPa respectively).^9^ The experimental techniques used by both differ to that used in this study, and involved pressurising the arterial specimens and measuring the oscillatory stress–strain relationship at various frequencies. Further differences may be expected in viscoelastic properties, as compared to this present study, as different types of arteries were analysed. The results of the femoral artery storage and loss moduli better match the coronary arteries measured during our study. Moreover, studies by Fischer and Llaurado^29^ have shown the collagen content of coronary arteries are similar to that of femoral arteries (percentage composition of collagen in dry defatted tissue; 47.9 ± 2.6 compared to 44.5 ± 1.4, respectively). It was noted, though, that coronary arteries had the highest collagen to elastin ratio.^29^

In this study we have noted a frequency-dependent trend for the storage modulus but not for the loss modulus. *Ergo*, as the heart rate increases, the LAD arterial wall is less able to store energy which is used for elastic recoil. This implies a change in the collagen-gel interaction leading to the storage of deformation energy within the tissue matrix with loading frequency. These deformations can involve elastic and plastic gel deformation and shearing on collagen fibrils.^37^,^39^ A previous study by Wang *et al.*, observed that above 10 Hz the elastic modulus of murine pulmonary arteries increased with frequency.^105^ In our current study we did not note an increase in moduli with frequency. The comparison is caveated, though, because there is no expectancy for the dynamic mechanical behaviour of a murine pulmonary and porcine coronary arteries to be parallel to each other. Further, Wang *et al.* characterised the frequency-dependent elastic modulus, as opposed to the dynamic viscoelasticity characterised in our current study. However, it is worth noting that between 1 and 10 Hz there was a decrease in elastic modulus in the study by Wang *et al.* demonstrating some consistency with findings from our current study.

Surface roughness of coronary arteries has not previously been quantified, however, *Ra* has been measured for other biological specimens, such as articular cartilage.^35^ Both the cartilage and endothelium have low frictional requirements. Cartilage values measured by Ghosh *et al*.,^35^ averaging 0.17 *µ*m using scanning electron microscopy, and varying from 0.08 to 0.11 *µ*m using atomic force microscopy, are lower than the values measured in this study. However, surface roughness measurements can vary between techniques used, as noted by Ghosh *et al*.^35^ For example, other studies of articular cartilage found its surface roughness to range from 0.08 *µ*m using laser profilometry, up to 1.60 *µ*m using stylus profilometry.^31^ Such values are comparable to the average mean of all measurements of 0.89 *µ*m (longitudinal) and 1.04 *µ*m (circumferential) in this present study. Of course, there is no reason for articular cartilage and blood vessels to have comparable surface roughness beyond both needing to be fairly smooth. However, no values are available for coronary arteries for comparison. Furthermore, it provides a comparison for another smoothed surface connective tissue, demonstrating a credible range for a biological material.

Computational models are useful to study coronary arteries. For example, they can be used to create patient specific models, as an alternative when an experiment is not possible, or to supplement experimental investigations.^47^ Viscoelastic properties of arteries have been found to be altered in patients with vascular diseases.^98^ Hence, finite element models could be created which incorporate viscoelastic properties, where variation of these mechanical properties could be used to study coronary artery disease.^98^ Surface properties of the endothelial surface can be seen to impact the blood flow and, therefore, a greater understanding of the surface roughness of coronary arteries can improve computational fluid dynamics modelling.^87^ Combined, *Ra* and viscoelasticity properties can progress fluid–structure interaction models; already used for cardiovascular modelling.^24^ This is beneficial as computer models have the potential to predict physiological functional interactions, how to better repair injuries, and improve diagnostics and treatment of disease.^50^

The surface roughness of novel biomaterials and surgical devices can be altered using surface modification techniques. The surface roughness measured in this study can be used as a standard to replicate natural surfaces^83^ through surface modification methods. Surface modification allows the bulk modulus of the material to remain unchanged, but with the additional benefit of being able to: increase the biocompatibility of materials;^40^,^66^,^72^,^74^,^111^ influence cell growth, alignment, viability and attachment;^27^,^30^,^46^,^57^,^62^,^70^,^91^ and increase patency rates by deterring thrombus formation.^20^,^40^,^84^ Briefly, modifications are created through removal techniques (sandblasting, anodisation, etching, lithography), addition techniques (coating, nanofibers, biomolecules), or a combination of both.^13^,^40^

New emerging biomaterials require a standard for surface roughness. The surface roughness properties of this study contribute to providing data for such a standard and a methodology for making further measurements. This would enable artificially created surfaces, such as textured materials and stents, and surfaces encouraging endothelialisation coverage,^56^,^73^,^93^,^109^ to be compared to healthy coronary arteries. Potentially this information could also be used to analyse disease. For example, similar to changes in the surface roughness of red blood cells with disease;^4^,^14^,^36^ or the correlation of stage of osteoarthritis, of human articular cartilage, to a fractal dimension.^88^

When measuring *Ra*_L_, the line along which the surface roughness was measured does not run perfectly parallel to the direction of the ridges. In fact, there appeared to be a more helical layout within blood vessels, which could be related to the spiral flow seen in other studies.^79^,^97^ Therefore, *Ra*_L_ would have peaks and troughs analogous to surface roughness measured circumferentially. The circumferential roughness was found to be higher than the longitudinal measurement for uncorrected surface roughness (*Ra*_CU_). This could be due to the greater prevalence of the ridges seen on the surface, which have been noted in previous qualitative studies.^15^,^26^

For corrected surface roughness (*Ra*_C_), no difference was found in the circumferential and longitudinal directions. The correction factor used in this study took measurements of the wet sample pre-processing. A limitation of the optical microscope is that it does not measure wet samples well, because water on the surface of the sample can distort images. It is possible, therefore, that *Ra*_C_ measurements taken pre-processing contain errors due to water gathering in the ridges seen on the sample surface. This would affect *Ra*_L_ measurements less as the variation would be seen more greatly circumferentially than longitudinally. For articular cartilage, processing causes the surface to increase in friction, probably due to the loss of proteins.^89^ However, despite the average roughness increasing, the fractal dimension was shown not to be effected by processing.^96^ Thus, *Ra*_CU_ and *Ra*_C_ likely provide results which represent outer limits of surface roughness when measured using an optical microscope. Thus, following tissue processing, *Ra*_C_ and *Ra*_CU_ may represent the lower and higher bounds of surface roughness for coronary arteries.

### Limitations

The fixation of tissue preserved the structure of samples and prevented degradation of tissue by cross-linking proteins.^45^,^52^,^81^ Processing is then completed by dehydrating the sample, ensuring that the specimen does not shrink and cause the surface to collapse due to the surface tension of water leaving the specimen.^28^ Although processing of tissue is useful for preserving the structure of samples, a limitation to this study are the structural changes which result through this process. Equation (6) was necessary to compensate for circumferential, but not radial, changes in *Ra*. The use of correction factors for quantitative measurements to compensate for tissue processing is well established having been used for changes in heart dimensions,^48^ volume of prostate cancer,^94^ and spatial dimension of brain tissue.^75^

The protocol for storing soft tissue by freezing used in the present study followed standard protocols used by other studies of porcine heart tissue.^22^,^23^,^25^,^77^ Clark, however, noted stiffening of vascular tissue when comparing frozen to fresh human aortic and mitral leaflets and chordae.^18^ It is noted, though, that there was extensive overlap in results from fresh and frozen specimens in Clark’s results. Further, storing other soft tissues at −20 °C have revealed no changes in mechanical properties including porcine liver,^106^ porcine aortic samples^85^ and murine tendons^38^ to name but a few. Moreover, neither repeated freeze–thaw cycles or extended frozen storage have been found to lead to more than minimal changes in biomechanical properties, for bone-patella tendon-bone soft tissue allografts^55^ and porcine aortic tissue,^85^ respectively. Instead, freezing temperature^38^ and method of freezing preservation^2^ may be of greater relevance. For this current study, an accepted protocol for storing fresh connective tissues −40 °C was followed,^25^ consistent with the recommendation of using a freezing, rather than refrigeration, protocol to maintain initial stress–strain behaviour of aortic tissue.^16^

## Conclusions

The following conclusions can be made of porcine LAD arteries:

- a frequency-dependent trend was observed for the storage modulus where, as the frequency was increased, the storage modulus decreased from (mean ± SD) 22.16 ± 8.75 MPa at 0.5 Hz to 17.75 ± 7.40 MPa at 10 Hz;
- the mean loss modulus was 2.10 ± 0.33 MPa, independent of frequency;
- the storage modulus was found to be much greater than the loss;
- storage and loss moduli did not vary along the length of the LAD artery;
- no significant difference was seen between the moduli results when measured at different extension ranges of 1–1.5 and 1.5–2 mm;
- the uncorrected surface roughness value measured circumferentially was greater than measured longitudinally (1.69 ± 0.75 *µ*m compared to 0.89 ± 0.27 *µ*m, respectively);
- for corrected surface roughness, there was no significant difference between the circumferential measurement compared to the longitudinal surface roughness (1.04 ± 0.46 *µ*m compared to 0.89 ± 0.27 *µ*m, respectively);
- for both uncorrected and corrected surface roughness, circumferential and longitudinal measurements did not vary along the length of the LAD artery;
- no relationship was found between viscoelastic properties and surface roughness.

Critically, though, this study demonstrates the feasibility of quantifying viscoelastic properties and the surface roughness of coronary arteries.
